# Supplementary material for: Hypertension management: experiences, wishes and concerns among older people—a qualitative study
Source: BMJ Open. 2019 Aug 18;9(8):e030742. doi: 10.1136/bmjopen-2019-030742 (PMC6701601; doi:10.1136/bmjopen-2019-030742)
Supplement: Supplementary data [file bmjopen-2019-030742supp001.pdf]

Supplementary file

Hypertension management: experiences, wishes and concerns among older people – a qualitative study

Table S1. Characteristics of participants

Gender	Age (years)	Multimorbidity [a]	Living location [b]	Educational level	Dependency [c]	Number of AHM	Total number of prescriptions [d]
**Female**	74	Yes	Small city	Middle	Independent	3	8*
**Male**	74	No	Village	High	Independent	2	2
**Female**	75	Yes	Small city	Unknown	Independent	1	3*
**Female**	75	No	Village	High	Independent	2	4
**Female**	77	Yes	Small city	Unknown	Independent	3	5
**Female**	79	Yes	Village	High	Independent	2	4
**Female**	80	Yes	Town	High	Independent	2	7*
**Male**	81	No	Small city	High	Independent	1	3
**Male**	82	Yes	City	Primary	Independent	3	7
**Male**	84	No	Village	High	Independent	3	4
**Male**	84	No	Village	Middle	ADL independent	2	6
**Female**	86	Yes	Town	High	Independent	2	4
**Female**	86	Yes	City	Middle	ADL independent	3	7
**Female**	87	No	Village	Middle	Independent	1	2
**Female**	93	No	City	Primary	ADL independent	2	3

AHM, antihypertensive medication; ADL, activities of daily living.
a. Multimorbidity (y/n), defined as having at least two chronic conditions, in addition to hypertension. Medical history based on self-report during interview.
b. City, >100,000 inhabitants; Small city, 10,000-100,000 inhabitants; Town 5,000-10,000 inhabitants; Village <5,000 inhabitants
c. Independent: able to take care of ADL and doing household chores/ run errands. ADL independent: able to take care of ADL, but dependent on doing household chores/ run errands.
d. * indicates the participant used a medication box, no one used medication rolls.
