# Supplementary material for: Hypertension management: experiences, wishes and concerns among older people—a qualitative study
Source: BMJ Open. 2019 Aug 18;9(8):e030742. doi: 10.1136/bmjopen-2019-030742 (PMC6701601; doi:10.1136/bmjopen-2019-030742)
Supplement: Author's manuscript [file bmjopen-2019-030742.draft_revisions.pdf]

BMJ Open is committed to open peer review. As part of this commitment we make the peer review history of every article we publish publicly available.

When an article is published we post the peer reviewers' comments and the authors' responses online. We also post the versions of the paper that were used during peer review. These are the versions that the peer review comments apply to.

The versions of the paper that follow are the versions that were submitted during the peer review process. They are not the versions of record or the final published versions. They should not be cited or distributed as the published version of this manuscript.

BMJ Open is an open access journal and the full, final, typeset and author-corrected version of record of the manuscript is available on our site with no access controls, subscription charges or pay-per-view fees (http://bmjopen.bmj.com).

If you have any questions on BMJ Open's open peer review process please email info.bmjopen@bmj.com

BMJ Open

Hypertension management: experiences, wishes and concerns among older people – a qualitative study

Journal:	*BMJ Open*
Manuscript ID	bmjopen-2019-030742
Article Type:	Research
Date Submitted by the Author:	29-Mar-2019
Complete List of Authors:	van Bussel, Emma; Amsterdam UMC, Department of Primary care and Medical Ethics<br>Reurich, Leony; Amsterdam UMC, Department of Primary care and Medical Ethics<br>Pols, A.; AMC, Department of Primary care and Medical Ethics<br>Richard, Edo; Academic Medical Centre, University of Amsterdam, Neurology; Neurology<br>Moll van Charante, Eric; Amsterdam UMC, AMC, Department of Primary care and Medical Ethics<br>Ligthart, Suzanne; Radbout university medical center
Keywords:	older people, PRIMARY CARE, conspiracy of silence, Hypertension management, shared decision-making

SCHOLARONE™
Manuscripts

Hypertension management: experiences, wishes and concerns among older people – a qualitative study

Emma F. van Bussel[1], L.H.G. (Leony) Reurich[1], A.J. (Jeannette) Pols[1], Edo Richard[2,3], Eric. P. Moll van Charante[1], Suzanne A. Ligthart[4]

1. Department of Primary care and Medical Ethics, Academic Medical Center Amsterdam, Meibergdreef 9, 1100 DD, Amsterdam, the Netherlands
2. Department of Neurology, Academic Medical Center Amsterdam, Meibergdreef 9, 1100 DD, Amsterdam, the Netherlands
3. Department of Neurology, Donders Centre for Brain, Behaviour and Cognition, Radboud University Medical Center, Geert Grooteplein 10, 6525 GA, Nijmegen, the Netherlands
4. Department of General Practice, Radboud University Medical Center, Geert Grooteplein 21, 6525 EZ Nijmegen, the Netherlands

Correspondence to: E.P. Moll Van Charante; Academic Medical Center Amsterdam, Meibergdreef 9, 1100 DD, Amsterdam, the Netherlands; Phone, +31-20-5667462; E-mail, e.p.mollvancharante@amc.uva.nl

For peer review only

Abstract

Objectives: Sixty-five percent of older people have hypertension, but little is known about their preferences and concerns regarding hypertension management. Guidelines on hypertension lack consensus on how to treat older people without previous cardiovascular disease. This asks for explicit consideration of patient preferences in decision making. Therefore, the aim of this study was to explore older peoples' experiences, preferences, concerns and perceived involvement regarding hypertension management.

Design: Qualitative interview study.

Setting: Participants were selected from 11 general practitioner (GP) practices in the Netherlands, and purposively sampled until data saturation was achieved. Semi-structured interviews were conducted, audio recorded and analyzed by two researchers using thematic analysis.

Participants: Fifteen community dwelling older people aged 74-93 years with hypertension and without previous cardiovascular disease participated.

Results: Interviewees rarely started the conversation about hypertension management themselves, although they did have concerns. Reasons for not discussing the subject included low priority of hypertension concerns, dependency on GPs or trust in GPs to make the right decision on their behalf. Also, interviewees anticipated regret of reducing medication, fearing vascular incidents. Interviewees would like to discuss tailoring treatment to their needs, deprescription of medication and ways to reduce side effects. They expected GPs to be more transparent on treatment effects.

Conclusion: Older people have little involvement in hypertension management, although they have several concerns. Since GPs are also known to be hesitant to bring up this subject, we signal a conspiracy of silence about antihypertensive medication. Through breaking this silence, GPs can facilitate shared decision-making on hypertension management and better tailored care.

Keywords: hypertension management, older people, primary care, shared decision-making conspiracy of silence

For peer review only

Strengths and limitations of this study:

- Interviews were performed at the participants' homes, enabling them to speak freely about their experiences, wishes and concerns.
- A diverse population of older persons was interviewed.
- Few participants experienced side effects of their medication.
- Information on decision making at the very start of hypertension treatment was limited, due to recall bias.

For peer review only - http://bmjopen.bmj.com/site/about/guidelines.xhtml

Funding
This research was supported by the Netherlands Organisation for Health Research and Development (grant number 839110003). The funders had no role in study design, methods, subject recruitment, data collection and analysis, interpretation of data, preparation of the manuscript or the decision to publish.

Competing interests
The Authors declare that there are no conflicts of interest.

For peer review only

For peer review only - http://bmjopen.bmj.com/site/about/guidelines.xhtml

Introduction

General practitioners consider hypertension management for older people challenging.[1] For people aged 70 years and over, primary prevention guidelines on cardiovascular disease lack consensus on (de)prescribing antihypertensive medication (AHM). Treatment decisions are further complicated by multi-morbidity, shorter life-expectancy, functional decline, and attenuating benefit-to-harm ratios.[2-5] These uncertainties underline the need for patient involvement in decision making, to tailor care.[5] General practitioners (GPs) wish to take into account patients' preferences and circumstances. However, in everyday practice these are not always optimally addressed and are generally not discussed.[1, 6, 7]

Little is known about older patients' involvement in and preferences on hypertension management in primary care. This is surprising since 65% of older people have hypertension and 82% of them are using antihypertensive medication.[8] Previous studies showed that older people wish to be involved in decision-making about their medical conditions, and expect their GP to consider their personal preferences, situation and concerns in decision-making.[9, 10] There is no reason to think this would be different for hypertension management.

Insight in older patients' experiences regarding hypertension management can support GPs in involving patients in decision making and thus in tailoring hypertension management. With this study we aimed to explore older peoples' experiences with AHM, how they perceive their involvement in hypertension management, and what their preferences and concerns regarding hypertension management are.

Methods

Participants

Community dwelling hypertensive persons aged 70 years or older were recruited from GP practices. We included participants without a history of cardiovascular disease (CVD) because people who experienced CVD may have different motivations for preventing future CVD and taking preventative medication, compared to people without CVD. To ensure diversity we purposively selected older persons based on age, gender, educational level, geographical region, urbanization, functional abilities and experiences with adverse effects of antihypertensive medication. GPs were asked to recruit a maximum of two patients per practice. All participants who agreed to have their contact details passed on to the researchers signed written informed consent and participated. A gift voucher (€10) was offered to participants as compensation for their time.

Data collection

Semi-structured interviews with use of a topic list were held at the participants' homes by LR, EB or both between January and March 2018. In 2018, LR and EB were both female GP trainees who had worked a year in a GP practice and with experience in qualitative and quantitative research. The interview started after introductions, small talk and reiterating the research topic and goal. All participants were interviewed on their views on hypertension, reasons to (not) treat it, the

For peer review only - http://bmjopen.bmj.com/site/about/guidelines.xhtml

experienced and desired role of the GP, and their own role in treatment decision making. The topic list was modified during the study period, as new themes arose during the first interviews. The interview topic list is presented in Table 1. Once data saturation was achieved (after 15 interviews) no new participants were recruited. Field notes were made after the interviews. The duration of the interviews ranged from 20 to 90 minutes.

Analysis

All interviews were audio recorded and transcribed verbatim. Qualitative analysis of the data was performed following the six phases of thematic analysis according to Braun and Clarke[11]:

1) First, two authors (LR and EB) familiarized themselves with the collected data by transcribing the interviews verbatim and repeatedly listening to and reading the data.
2) LR and EB coded the first six interviews independently by systematically going through the data, creating initial codes. After comparing three independently coded interviews, codes were merged. The next three interviews were coded independently, and compared, resulting in a new set of codes. The other interviews were coded by one author using these codes and checked by the other. The coding was compared and discussed until agreement was found.
3) Identified codes were sorted into potential themes which were discussed by the authors (LR, EB, JP, EMC, SL) until consensus about potential themes was reached.
4) Potential themes were reviewed, and thematic maps were made. The collected data was re-read to make sure the thematic map was representing the data set. Whether data saturation was reached was discussed.
5) The resulting themes were refined and a narrative of the found data was considered and discussed by LR, EB, JP, EMC, SL.
6) Final analysis was performed, and illustrative examples were selected in order to answer the research question and compare our analysis to existing literature.

For coding and analysis MAXQDA Plus 12 (version 12.2.1) was used. Reporting of our study is in accordance with the consolidated criteria for reporting qualitative research (COREQ).[12]

Ethical considerations

Official approval of this study was waived by the Medical Ethics Review Committee of the Academic Medical Centre in Amsterdam (reference W18_004#18.015), the Netherlands.

Patient and public involvement

There were no patients involved in the development of the research question, the design recruitment to or conduct of the study. However, this study was specifically designed to bring together the perceptions of hypertensive older patients, to inform hypertension management in general practice.

For peer review only - http://bmjopen.bmj.com/site/about/guidelines.xhtml

Results

In total fifteen individuals aged 74-93 years (mean 81) from eleven different GP practices were interviewed. Two lived in senior apartments, all interviewees were living independently. Three received help with groceries and used walking aids. Participants had 3 to 8 (median 4) prescriptions, with 1 to 3 (median 2) types of antihypertensive medication, and level of education ranged from primary school to higher education.(Table S1) Partners of three interviewees were present in the same room during the interview, and occasionally confirmed, contradicted or supplemented the interviewee. Two interviewees had experienced side effects from antihypertensive medication (AMH).

Hypertension management is not discussed

Interviewees had regular check-ups for their hypertension management, often with a practice nurse. During those visits, management was discussed superficially. In general, participants did not discuss their concerns regarding hypertension management with their GPs or felt barriers to do so. When directly asked why, interviewees expressed that there was no need to discuss the subject, or that they experienced barriers to discuss issues on hypertension with their GP. During the interviews however, all participants reported unexpressed concerns. In general, they expected (and waited for) the GP to start the conversation about their preferences and concerns regarding hypertension management. One participant who felt reluctant taking medication reported feeling extremely relieved when her GP suggested to reduce her antihypertensive medication.

'Then she [the GP] said: "why don't we try to reduce the medication?" At that moment I could hug her, I would never dare to ask this myself (…), but if she wants to!' (P6, 86-year-old woman, 2 AHM)

There were four key reasons why older persons did not start the conversation about hypertension management with their GP, and four reasons why they desired such a discussion.

Reasons to not discuss needs and preferences regarding hypertension management

<u>Hypertension is not a priority</u>

When participants experience no symptoms of hypertension or side effects of treatment, they generally did not seem to bother much about hypertension or antihypertensive medication (AHM). Interviewees felt hypertension is part of normal aging, and other diseases or circumstances were more important to them. Taking AHM on a daily basis was part of their routine and did not seem to burden these participants.

'I am not bothered by it [hypertension], so I never think about it. I just never think about it' (P7, 86-year-old woman, 3 AHM)

<u>Dependency on the GP</u>

Fear of jeopardizing the relation with the GP, on whose care they relied for all other (medical) problems, was a barrier to express concerns regarding AHM.

For peer review only - http://bmjopen.bmj.com/site/about/guidelines.xhtml

'You feel dependent on them. You cannot fight with them, because what can you do, it is not easy to change or find another GP (…) so your dependency is bigger than I anticipated.' (P5, 81-year-old man, 1 AHM)

Also, the perceived authority of the GP formed a barrier to discuss any doubts about AHM. Some interviewees felt that it would be inappropriate to question the GP's judgement.

'I don't want to do that [discuss reducing medication], because then I disregard my GP's advice, my GP expects me to follow her advice' (P1, 82-year-old man, 3 AHM)

The GP knows best

Another reason to remain silent was trust in the GP and his or her expertise. Interviewees were convinced that the GP would continuously monitor their medication and trusted him to start the conversation if this was indicated.

'I just assume that if he thinks I need another pill, that he knows what is best for me' (P2, 77-year-old woman, 3 AHM)

The long-term relation with the GP enforced this trust in the GP's expertise.

'I thought, well, the doctor is probably right, who am I, I didn't study for it, so he probably knows, because he knows me already for a long time'. (P2, 77-year-old woman, 3 AHM)

Interviewees found it difficult to truly understand the consequences of risk (reduction) for their personal situation and to interpret that information. This was a barrier to making decisions and discussing doubts with their GP. The uncertainty made interviewees rely on the advice of their GP; the expert.

'I would like to know [statistics on risk reduction], but I would like to get an answer which says something about my own risk, but there is no such answer, that makes it difficult.' (P5, 81-year-old man, 1 AHM)

Anticipated regret and avoiding responsibilisation

Interviewees were aware of the threats of hypertension. They feared CVD, particularly cerebrovascular accidents, since it often comes with disability, loss of independence and loss of social contacts. One of the strongest motivators not to express concerns about drug treatment was anticipated regret of developing CVD, when reducing medication.

'I don't want to have a stroke (…). I have worked as an activity coordinator in nursing homes. I have seen the results of strokes, I don't want to experience that.' (P10, 79-year-old female, 2 AHM)

In addition, actively initiating change in hypertension management felt like taking responsibility for the outcome. Following doctor's advice, and ignoring concerns about hypertension management was a way to avoid active decision making and responsibility for one's own situation.

For peer review only - http://bmjopen.bmj.com/site/about/guidelines.xhtml

‘I am not going to be stubborn, because if something would happen, it would be my own fault.’ (P2, 77-year-old woman, 3 AHM)

In the same line, fear of what might happen kept interviewees from wanting to learn about the details and risks of their condition.

‘No, I don’t want that [more explanation of the doctor about AHM], no. I mean, if he explains to me what will happen if I don’t take them [medication] I would feel uneasy, I think.’ (P7, 86-year-old woman, 3 AHM)

Concerns and preferences regarding hypertension management

Wish to reduce antihypertensive medication (AHM)

Even if they did not discuss this with their GP, when asked the interviewees showed interest in reducing medication and had questions about their use of medication. First, there was a wish to minimize overall medication use. Second, participants wondered whether (some of) the medication could be deprescribed, after a certain period of adequately controlled blood pressure. They were curious to find out if hypertension would reappear after stopping medication.

‘My blood pressure is always good, my question is, is that because of the medication? Or maybe I do not need the medication?’ (P14, 75-year-old woman, 1 AHM))

Wish to consider one’s age and situation

Taking into account one’s age and situation when deciding on continuation of AHM was considered important. For instance, a participant expressed the concern that his GP would not (sufficiently) adjust his blood pressure levels to his older age:

‘Sometimes I feel that they think you are still a young guy, because you have to reach certain [blood pressure] levels, which might not be relevant anymore for older people.’ (P4, 84-year-old man, 3 AHM)

Some participants thought that relatively high levels of stress in the past might have contributed to increased blood pressure levels at the start of their treatment and wondered whether improvements in their wellbeing could have had beneficial effects, allowing for deprescription of AHM.

‘”Your blood pressure is too high”, well that was based on certain levels, it is possible, I don’t have knowledge about that (…), but I keep thinking that it was a particular moment’. (P14, 75-year-old woman, 1 AHM)

Side effects

Fatigue, fogginess, dizziness, staggering gait, polyuria, nausea, burning sensation in the stomach and reduced exercise tolerance were side effects of AHM that participants reported. These side effects reduced quality of life and resulted in the wish to re-evaluate the benefits and harms of continuing or stopping AHM. For some, this was a reason to discuss hypertension treatment with their GP.

For peer review only - http://bmjopen.bmj.com/site/about/guidelines.xhtml

'If it stays like it is at the moment, I think I will accept it. But that burn in your stomach and the feeling that you have to throw up, I don't like that, that was not acceptable for me. It needs to be in my opinion within reason, these side effects. If it becomes too much, I won't accept it. I would then say: "I don't want that drug anymore".' (P15, 74-year-old woman, 2 AHM)

Lack of transparency in GPs' considerations

The interviewees who experienced side effects of AHM learned about the uncertainty of their GP regarding hypertension management, during their struggle to find a balance between prevention of CVD and side effects. Despite the knowledge that GPs do not know the optimal blood pressure targets in older age, and thus the right treatment, one of them felt reluctant to discuss his own doubts with the GP. Still, he would have appreciated it if his GP had shown and shared more of his uncertainty, to make room for alternative outcomes (instead of blood pressure target as the only goal).

'They really don't know. They have statistics, but whether you meet those or not, they don't know, they just don't know. I think they should be more transparent about that.' (P5, 81-year-old male, 1 AHM)

Uncertainty about implications of potential choices

Overarching the aforementioned themes is the uncertainty of implications of choices regarding hypertension management. Interviewees were ambivalent about deciding which of their concerns and preferences were most important, and should be acted upon.

'It [taking antihypertensive medication] is a compromise. (…) Of course, if it would be better [to take more medication] and if it lowers my risk I would accept it. I wouldn't be happy about it. I would be reluctant [to take medication] but it would be necessary, it would be better because you would lower your risks.' (P3, 74-year-old woman, 2 AHM)

The final thematic map is shown in Figure 1.

Discussion

Older people do not feel sufficiently involved in decision making about hypertension management in general practice, while they do have many unexpressed concerns. Reasons not to raise these concerns are the low priority compared to other complaints or diseases, being dependent on their GP, trust in their GP to make the right decision on their behalf, and anticipated regret of incident cardiovascular disease (CVD) in absence of treatment. Participants could benefit from overcoming these barriers but wait for their GP to bring up the subject. They often have the wish to reduce medication, to tailor management to their individual situation, to avoid side effects and to receive transparent information on expected treatment effects. Lastly, participants experienced ambivalence

For peer review only - http://bmjopen.bmj.com/site/about/guidelines.xhtml
For peer review only

regarding their concerns and preferences in hypertension management, hampering their perceived capacity for decision making on their own situation.

Strengths of this study include that interviews were performed at the participants' homes, by researchers who were independent of their GPs. This way we enhanced trust and enabled a diverse population of older persons to speak freely about their experiences, wishes and concerns.
Decision making at the very start of hypertension treatment was hard to recall, limiting the information on treatment initiation. Yet, a large proportion of Dutch older adults have long term hypertension and our results apply to this group.[8] With only two participants (recently) experiencing side effects of antihypertensive medication (AHM), this groups representation was small in our study, which may have limited our findings. It was difficult to include persons with side effects from AHM, since most older persons were using AHM for a long time without any problems, with difficulty to recall past episodes of problems or reluctance to discuss this with their GP. It is reported that in adults up to 70 years of age, 85% of those starting AHM experienced side effects,[13] while of long-term AHM users (up to 84 years), 20% reported side effects.[14]
Although we aimed to include people without a history of CVD, two participants probably had had CVD (P9 and P15) judging from the interviews and their medication list (acenocoumarol, acetylsalicylic acid). When selecting participants, we relied on the GP to have assessed the inclusion criteria. However, in our study opinions did not differ from participants with definite absence of CVD in their medical history, particularly not on motivation for preventing future CVD or willingness to use preventative medication.
Our results provide insight into experiences and views of older persons with hypertension, that may apply to other developed countries. Although the next generation of older people may have more difficulty with accepting authority in GPs and less problems with speaking up about their concerns, the complexity of decisions in hypertension management is not likely to change.

<u>A conspiracy of silence</u>

There appears to be a conspiracy of silence, in which both the older patient and the GP do not express their concerns regarding antihypertensive treatment. From previous studies it is known that GPs feel that they hardly involve older persons in decisions on hypertension management.[1, 6, 7] Reasons are time constraints, automated prescriptions, negative emotional impact on patients and anticipated regret in case of cardiovascular events.[6] This study showed that older people too, have the impression of no or minimal involvement in their hypertension management in primary care. From prior studies little is known about (the perceived) involvement of older people in their hypertension management.[15] This contrasts the wish for greater patient involvement in hypertension management among both GPs and older patients.[1, 6, 7]

<u>Reasons to remain silent</u>

There is uncertainty and ambivalence in prioritizing preferences and concerns regarding antihypertensive medication use among older people in our study. Ambivalence has previously been shown in qualitative studies among older people. Antihypertensive medication can provide a feeling of security regarding prevention of CVD, but older patients also worry about side effects and long-

For peer review only - http://bmjopen.bmj.com/site/about/guidelines.xhtml

term adverse effects of taking chronic medication,[16-18] are cautious to take preventative medication and prefer minimizing medication-use.[17, 19] Concerns are generally outweighed by the perceived necessity of treatment and their GP's recommendation.[19, 20] In our study side effects were the only reason to discuss alternatives with the GP.

Dependency on the GP was a major barrier that is also recognized in shared decision-making (SDM) in other fields. Patients are in a vulnerable position, and can feel too dependent to bring up concerns in interaction with the doctor.[21] Other barriers to active involvement in decisions were reluctance to bear responsibility for cardiovascular outcomes and anticipated regret, also known from SDM in other fields.[21] It was previously shown that anticipated regret has stronger associations with health behavior than other anticipated negative emotions and risk appraisals.[22]

In our study hypertension and hypertension management were generally given low priority. When taking AHM, participants felt safe in terms of preventing CVD morbidity and mortality. However, older people both overestimate the risks of hypertension and the benefits of medication. E.g. hypertensive people with a mean age of 73 year estimated their stroke risk to be 40% within 5 years, and expected over 50% stroke risk reduction from AHM, while in fact 5 year risk of cardiovascular events for this group is 5-20% and stroke risk reduction from AHM is 24-42%.[23-25] After being shown the actual risks and benefits, a quarter of 75 older patients with hypertension participating in a qualitative study became uncertain about antihypertensive medication use, or would decline to take it.[23] Trust in GPs and AHM seems high, but also unrealistic so participants' choices are often based on false, or at least incomplete information. Concerns about taking medication, side effects and tailoring to the individual situation, could receive different prioritization if patients would be better informed and if GPs would be more transparent on their uncertainty.

Breaking the silence

It is important to break the silence since older patients generally wish to be involved in decision-making about their medical issues, despite the abovementioned reasons to remain silent.[9] GPs also value patient involvement in decision making, especially since guidelines leave room for discussion.[6, 26] By breaking the silence, GPs could guide older patients in their ambivalent feelings about AHM, and improve personalized care. For example, once started, lifelong use of AHM is the current standard of care. However, a systematic review showed that after 2 years of withdrawal of AHM, hypertension did not return in 26% of patients (mean age 41 to 76 years). This justifies an AHM withdrawal attempt in well controlled patients, if they wish to do so.[27] In addition, more transparency could reduce misinterpretations and lower the threshold to start discussing factors important to patients in hypertension management.

Heterogeneity exists in how older people want to be involved in decision-making. Older patients with multi-morbidity and healthcare professionals agreed that SDM in older patients requires a continuous dialogue between professional and patient.[28] Contextual factors for receiving information, such as having enough time and having a good relationship with professionals involved, are considered of great importance.[29] Elements in decision making unique to older people are the opportunity for input from trusted others and discussion of decisions' impacts on patients daily lives.[30]

For peer review only - http://bmjopen.bmj.com/site/about/guidelines.xhtml

Older persons generally do not start the dialogue themselves, they wait for their GP to do so. In our study, and also in SDM in other fields of medicine, older patients experience barriers to participate in decision making about their care.[31] Since most barriers are related to the GP, including the authority ascribed to them as well as time constraints, we recommend that GPs take the initiative to break the silence.[20]

Implication for practice

To break the conspiracy of silence, GPs should explicitly and repeatedly discuss hypertension treatment with their patients on chronic antihypertensive medication. This is the only way to come to true shared decision-making about hypertension treatment and may lead to adaptation and potentially cessation of antihypertensive medication in those for whom the benefits not clearly outweigh the burden.

For peer review only

For peer review only - http://bmjopen.bmj.com/site/about/guidelines.xhtml

Ethical approval

Official approval of this study was waived by the Medical Ethics Review Committee of the Academic Medical Centre in Amsterdam (reference W18_004#18.015), the Netherlands.

Financial disclosure

No financial disclosures were reported by the authors of this manuscript.

Acknowledgements

We sincerely thank all individuals who participated in this qualitative study, and all general practitioners and their assistants who selflessly recruited participants.

Author contributions

All authors contributed to the conception or design of the work. EB and LR performed the interviews. EB, LR, JP, EMC and SL contributed to the acquisition, analysis, or interpretation of data for the work. EB drafted the manuscript. All authors critically revised the manuscript and gave final approval and agree to be accountable for all aspects of work ensuring integrity and accuracy.

Data sharing statement

Data underlying our findings cannot be made publicly available for ethical reasons; public availability would compromise our participants' privacy. Data requests may be sent to the corresponding author at e.p.mollvancharante@amc.uva.nl

Licence statement

I, the Submitting Author has the right to grant and does grant on behalf of all authors of the Work (as defined in the below author licence), an exclusive licence and/or a non-exclusive licence for contributions from authors who are: i) UK Crown employees; ii) where BMJ has agreed a CC-BY licence shall apply, and/or iii) in accordance with the terms applicable for US Federal Government officers or employees acting as part of their official duties; on a worldwide, perpetual, irrevocable, royalty-free basis to BMJ Publishing Group Ltd ("BMJ") its licensees and where the relevant Journal is co-owned by BMJ to the co-owners of the Journal, to publish the Work in BMJ Open and any other BMJ products and to exploit all rights, as set out in our licence.

The Submitting Author accepts and understands that any supply made under these terms is made by BMJ to the Submitting Author unless you are acting as an employee on behalf of your employer or a postgraduate student of an affiliated institution which is paying any applicable article publishing charge ("APC") for Open Access articles. Where the Submitting Author wishes to make the Work available on an Open Access basis (and intends to pay the relevant APC), the terms of reuse of such Open Access shall be governed by a Creative Commons licence – details of these licences and which Creative Commons licence will apply to this Work are set out in our licence referred to above.

For peer review only - http://bmjopen.bmj.com/site/about/guidelines.xhtml

References

1. Jansen J, McKinn S, Bonner C, et al. General Practitioners' Decision Making about Primary Prevention of Cardiovascular Disease in Older Adults: A Qualitative Study. PLoS One. 2017;12(1):e0170228.
2. Perk J, De Backer G, Gohlke H, et al. European guidelines on cardiovascular disease prevention in clinical practice (version 2012) : the fifth joint task force of the European society of cardiology and other societies on cardiovascular disease prevention in clinical practice (constituted by representatives of nine societies and by invited experts). Int J Behav Med. 2012;19(4):403-88.
3. Streit S, Verschoor M, Rodondi N, et al. Variation in GP decisions on antihypertensive treatment in oldest-old and frail individuals across 29 countries. BMC Geriatr. 2017;17(1):93.
4. Weiss J, Freeman M, Low A, et al. Benefits and Harms of Intensive Blood Pressure Treatment in Adults Aged 60 Years or Older: A Systematic Review and Meta-analysis. Ann Intern Med. 2017;166(6):419-29.
5. Jansen J, McKinn S, Bonner C, et al. Systematic review of clinical practice guidelines recommendations about primary cardiovascular disease prevention for older adults. BMC Fam Pract. 2015;16:104.
6. van Middelaar T, Ivens SD, van Peet PG, et al. Prescribing and deprescribing antihypertensive medication in older people by Dutch general practitioners: a qualitative study. BMJ Open. 2018;8(4):e020871.
7. Clinch M, Benson J. Making information 'relevant': general practitioner judgments and the production of patient involvement. Soc Sci Med. 2013;96:104-11.
8. Nwankwo T, Yoon SS, Burt V, Gu Q. Hypertension among adults in the United States: National Health and Nutrition Examination Survey, 2011-2012. NCHS Data Brief. 2013(133):1-8.
9. Bastiaens H, Van Royen P, Pavlic DR, et al. Older people's preferences for involvement in their own care: a qualitative study in primary health care in 11 European countries. Patient Educ Couns. 2007;68(1):33-42.
10. Rotar-Pavlic D, Svab I, Wetzels R. How do older patients and their GPs evaluate shared decision-making in healthcare? BMC Geriatr. 2008;8:9.
11. Braun VC, V. . Using thematic analysis in psychology. . In: Clarke V, editor. Qualitative Research in Psychology2006. p. 77-101.
12. Tong A, Sainsbury P, Craig J. Consolidated criteria for reporting qualitative research (COREQ): a 32-item checklist for interviews and focus groups. Int J Qual Health Care. 2007;19(6):349-57.
13. Tedla YG, Bautista LE. Drug Side Effect Symptoms and Adherence to Antihypertensive Medication. Am J Hypertens. 2016;29(6):772-9.
14. Bardage C, Isacson DG. Self-reported side-effects of antihypertensive drugs: an epidemiological study on prevalence and impact on health-state utility. Blood Press. 2000;9(6):328-34.
15. Butterworth JE, Campbell JL. Older patients and their GPs: shared decision making in enhancing trust. Br J Gen Pract. 2014;64(628):e709-18.
16. Moen J, Bohm A, Tillenius T, et al. "I don't know how many of these [medicines] are necessary.." - a focus group study among elderly users of multiple medicines. Patient Educ Couns. 2009;74(2):135-41.
17. Benson J, Britten N. Patients' decisions about whether or not to take antihypertensive drugs: qualitative study. BMJ. 2002;325(7369):873.
18. Clyne B, Cooper JA, Boland F, et al. Beliefs about prescribed medication among older patients with polypharmacy: a mixed methods study in primary care. Br J Gen Pract. 2017;67(660):e507-e18.
19. Gale NK, Greenfield S, Gill P, et al. Patient and general practitioner attitudes to taking medication to prevent cardiovascular disease after receiving detailed information on risks and benefits of treatment: a qualitative study. BMC Fam Pract. 2011;12:59.

For peer review only - http://bmjopen.bmj.com/site/about/guidelines.xhtml

20. Modig S, Kristensson J, Troein M, et al. Frail elderly patients' experiences of information on medication. A qualitative study. BMC Geriatr. 2012;12:46.
21. van Laarhoven HW, Henselmans I, de Haes JH. To treat or not to treat: who should decide? Oncologist. 2014;19(4):433-6.
22. Brewer NT, DeFrank JT, Gilkey MB. Anticipated regret and health behavior: A meta-analysis. Health Psychol. 2016;35(11):1264-75.
23. Cranney M, Warren E, Walley T. Hypertension in the elderly: attitudes of British patients and general practitioners. J Hum Hypertens. 1998;12(8):539-45.
24. Ligthart SA, Moll van Charante EP, Van Gool WA, Richard E. Treatment of cardiovascular risk factors to prevent cognitive decline and dementia: a systematic review. Vasc Health Risk Manag. 2010;6:775-85.
25. Beer C, Alfonso H, Flicker L, et al. Traditional risk factors for incident cardiovascular events have limited importance in later life compared with the health in men study cardiovascular risk score. Stroke. 2011;42(4):952-9.
26. Wetzels R, Geest TA, Wensing M, et al. GPs' views on involvement of older patients: an European qualitative study. Patient Educ Couns. 2004;53(2):183-8.
27. van der Wardt V, Harrison JK, Welsh T, et al. Withdrawal of antihypertensive medication: a systematic review. J Hypertens. 2017;35(9):1742-9.
28. van de Pol MH, Fluit CR, Lagro J, et al. Expert and patient consensus on a dynamic model for shared decision-making in frail older patients. Patient Educ Couns. 2016;99(6):1069-77.
29. Robben S, van Kempen J, Heinen M, et al. Preferences for receiving information among frail older adults and their informal caregivers: a qualitative study. Fam Pract. 2012;29(6):742-7.
30. Price EL, Bereknyei S, Kuby A, et al. New elements for informed decision making: a qualitative study of older adults' views. Patient Educ Couns. 2012;86(3):335-41.
31. Bane C, Hughes CM, Cupples ME, McElnay JC. The journey to concordance for patients with hypertension: a qualitative study in primary care. Pharm World Sci. 2007;29(5):534-40.

For peer review only

For peer review only - http://bmjopen.bmj.com/site/about/guidelines.xhtml

Table 1. Interview topic list

**Topic**	Subtopic
**Participant characteristics**	Demographics, including medical history and medication use
**Hypertension and antihypertensive medication**	Frequency of hypertension visits at the GP practice
	Topics discussed during hypertension visits at the GP practice: medication use, side effects, lifestyle.
	Experience with hypertension, antihypertensive medication (use), and lifestyle (changes) since hypertension was first identified.
	Attitudes towards hypertension, antihypertensive medication (use), lifestyle, and necessity of treatment
	Experiences with and attitudes towards deprescribing antihypertensive medication
**Shared decision-making**	Received information on hypertension, risk and risk reduction from antihypertensive medication and lifestyle during (hypertension) visits at the GP practice
	Experience of being involved in decision making and receiving tailored information and care.
	Views on involvement and tailoring information and care by GP
	Experience of trust in the GP and in hypertension management in primary care
	Experiences of GPs' uncertainties in hypertension management in older persons
	Experience of and views on necessity, barriers and facilitators to discuss hypertension management with the GP
**Personal situation and preferences**	Experience of and views on consideration of personal circumstances and age in decision making in hypertension management
	Experienced effects of one's personal situation on views on hypertension and its management
	Experienced prioritization of facets of hypertension and its management
	Experienced effect of chronic hypertension and long-term medication use on views on hypertension and its management
**Future perspectives**	Wish to change things regarding hypertension management: lifestyle, antihypertensive medication use, or involvement in decision making with the GP.

GP, general practitioner

For peer review only

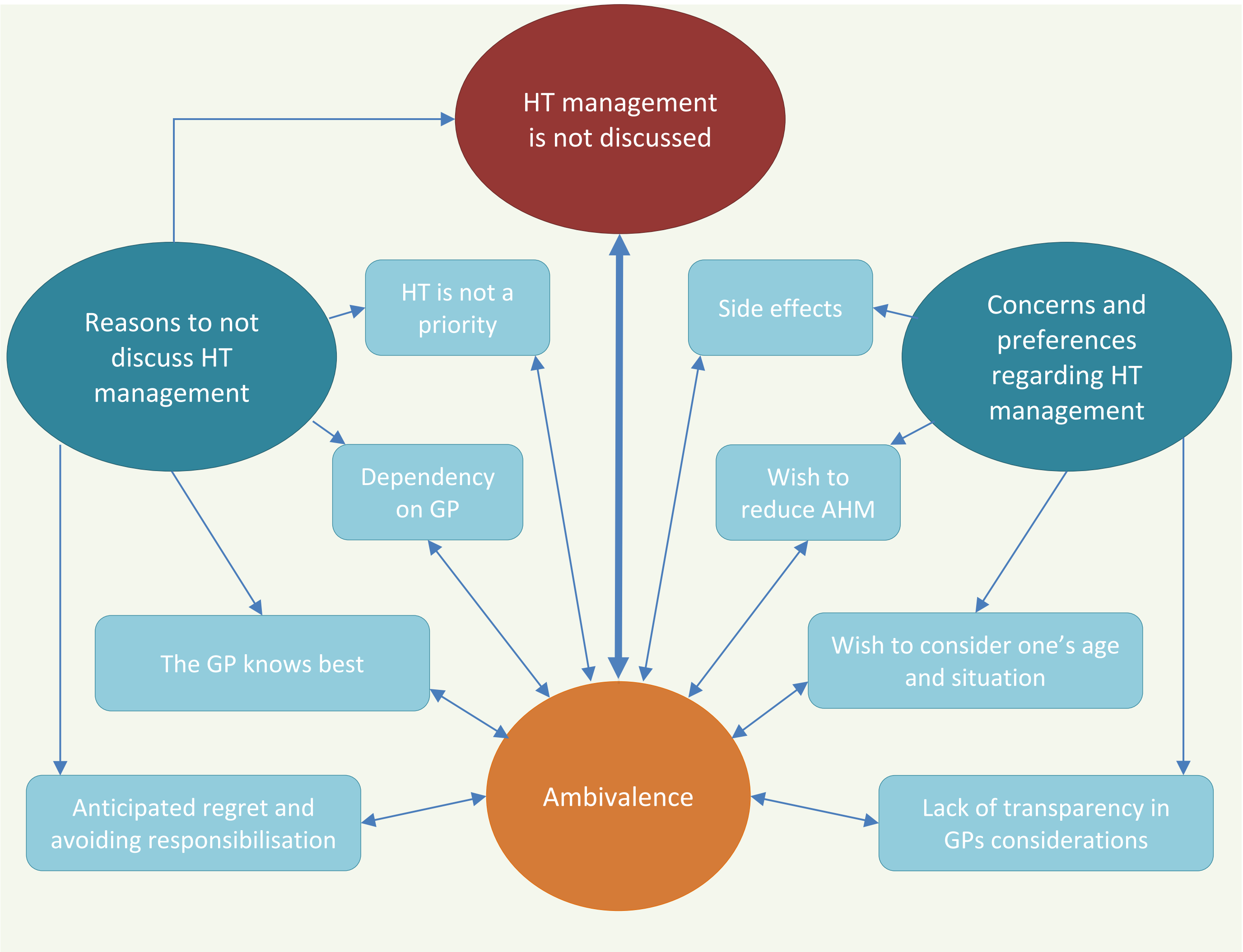

Figure 1. Final thematic map.

AHM, antihypertensive medication; GP, general practitioner; HT, hypertension.

Appendix

Hypertension management: experiences, wishes and concerns among older people – a qualitative study

Table S1. Characteristics of participants

**Participant number** [a]	Gender	Age (years)	Medical history [b]	Living location	Educational level	Dependency [c]	Number of AHM	Total number of prescriptions [d]
**P1**	Male	82	T2DM, heart failure	City	Primary	Independent	3	7
**P2**	Female	77	Angina pectoris, lower back pain	City	Unknown	Independent	3	5
**P3**	Male	74	none	Town	Higher	Independent	2	2
**P4**	Male	84	none	Town	Higher	Independent	3	4
**P5**	Male	81	Atrial fibrillation	Small city	Higher	Independent	1	3
**P6**	Female	86	Breast cancer, hyperthyroidism	Town	Higher	Independent	2	4
**P7**	Female	86	Chronic dermal infection, T2DM	City	Middle	ADL independent	3	7
**P8**	Female	75	none	Town	Higher	Independent	2	4
**P9**	Male	84	Total hip replacement both sides	Town	Middle	ADL independent	2	6
**P10**	Female	79	Cerebral palsy following encephalitis, knee osteoarthritis	Town	Higher	Independent	2	4
**P11**	Female	87	Atrial fibrillation	Town	Middle	Independent	1	2
**P12**	Female	93	Macular degeneration	City	Primary	ADL independent	2	3
**P13**	Female	80	Arthrosis, muscle dystrophy, tiredness	Town	Higher	Independent	2	7*
**P14**	Female	75	Breast cancer, neuropathic pains	Small city	Unknown	Independent	1	3*
**P15**	Female	74	Cardiac surgery, uterus surgery, hypothyroidism, lower back pain	Small city	Middle	Independent	3	8*

AHM, antihypertensive medication; ADL, activities of daily living; T2DM, type 2 diabetes mellitus.
a. Participants numbered based on interview date, with P1 being the first interview
b. in addition to hypertension, medical history based on self-report during interview
c. Dependency: independent, independent of others; ADL independent, ADL independent, but depends on others for cleaning the home and/or groceries; na, not applicable
d. * indicates the participant used a medication box, no one used medication rolls.

For peer review only

COREQ (COnsolidated criteria for REporting Qualitative research) Checklist

A checklist of items that should be included in reports of qualitative research. You must report the page number in your manuscript where you consider each of the items listed in this checklist. If you have not included this information, either revise your manuscript accordingly before submitting or note N/A.

Topic	Item No.	Guide Questions/Description	Reported on Page No.
**Domain 1: Research team and reflexivity**			
Personal characteristics			
Interviewer/facilitator	1	Which author/s conducted the interview or focus group?	4
Credentials	2	What were the researcher's credentials? E.g. PhD, MD	4,5
Occupation	3	What was their occupation at the time of the study?	4,5
Gender	4	Was the researcher male or female?	4,5
Experience and training	5	What experience or training did the researcher have?	4,5
Relationship with participants			
Relationship established	6	Was a relationship established prior to study commencement?	4,5
Participant knowledge of the interviewer	7	What did the participants know about the researcher? e.g. personal goals, reasons for doing the research	NA
Interviewer characteristics	8	What characteristics were reported about the inter viewer/facilitator? e.g. Bias, assumptions, reasons and interests in the research topic	NA
**Domain 2: Study design**			
Theoretical framework			
Methodological orientation and Theory	9	What methodological orientation was stated to underpin the study? e.g. grounded theory, discourse analysis, ethnography, phenomenology, content analysis	5
Participant selection			
Sampling	10	How were participants selected? e.g. purposive, convenience, consecutive, snowball	4
Method of approach	11	How were participants approached? e.g. face-to-face, telephone, mail, email	4
Sample size	12	How many participants were in the study?	6
Non-participation	13	How many people refused to participate or dropped out? Reasons?	4
Setting			
Setting of data collection	14	Where was the data collected? e.g. home, clinic, workplace	4
Presence of non-participants	15	Was anyone else present besides the participants and researchers?	4,5
Description of sample	16	What are the important characteristics of the sample? e.g. demographic data, date	6
Data collection			
Interview guide	17	Were questions, prompts, guides provided by the authors? Was it pilot tested?	4,5
Repeat interviews	18	Were repeat inter views carried out? If yes, how many?	na
Audio/visual recording	19	Did the research use audio or visual recording to collect the data?	4,5
Field notes	20	Were field notes made during and/or after the inter view or focus group?	4,5
Duration	21	What was the duration of the inter views or focus group?	4,5
Data saturation	22	Was data saturation discussed?	5
Transcripts returned	23	Were transcripts returned to participants for comment and/or	na

For peer review only

Topic	Item No.	Guide Questions/Description	Reported on Page No.
		correction?	
**Domain 3: analysis and findings**			
Data analysis			
Number of data coders	24	How many data coders coded the data?	5
Description of the coding tree	25	Did authors provide a description of the coding tree?	na
Derivation of themes	26	Were themes identified in advance or derived from the data?	5
Software	27	What software, if applicable, was used to manage the data?	5
Participant checking	28	Did participants provide feedback on the findings?	5
Reporting			
Quotations presented	29	Were participant quotations presented to illustrate the themes/findings? Was each quotation identified? e.g. participant number	6 - 9
Data and findings consistent	30	Was there consistency between the data presented and the findings?	6 - 9
Clarity of major themes	31	Were major themes clearly presented in the findings?	6 - 9
Clarity of minor themes	32	Is there a description of diverse cases or discussion of minor themes?	6-9

Developed from: Tong A, Sainsbury P, Craig J. Consolidated criteria for reporting qualitative research (COREQ): a 32-item checklist for interviews and focus groups. *International Journal for Quality in Health Care*. 2007. Volume 19, Number 6: pp. 349 – 357

Once you have completed this checklist, please save a copy and upload it as part of your submission. DO NOT include this checklist as part of the main manuscript document. It must be uploaded as a separate file.

For peer review only

Hypertension management: experiences, wishes and concerns among older people – a qualitative study

Journal:	*BMJ Open*
Manuscript ID	bmjopen-2019-030742.R1
Article Type:	Research
Date Submitted by the Author:	24-Jul-2019

Complete List of Authors:	van Bussel, Emma; Amsterdam UMC, Department of Primary care and Medical Ethics<br>Reurich, Leony; Amsterdam UMC, Department of Primary care and Medical Ethics<br>Pols, A.; AMC, Department of Primary care and Medical Ethics<br>Richard, Edo; Academic Medical Centre, University of Amsterdam, Neurology; Neurology<br>Moll van Charante, Eric; Amsterdam UMC, AMC, Department of Primary care and Medical Ethics<br>Ligthart, Suzanne; Radbout university medical center, Department of Primary and Community Care

**Primary Subject Heading**:	Qualitative research
Secondary Subject Heading:	Patient-centred medicine, General practice / Family practice
Keywords:	older people, PRIMARY CARE, conspiracy of silence, Hypertension management, shared decision-making

SCHOLARONE™
Manuscripts

Hypertension management: experiences, wishes and concerns among older people – a qualitative study

Emma F. van Bussel[1], Leony L.H.G. Reurich[1], A. Jeannette Pols[1], Edo Richard[2,3], Eric. P. Moll van Charante[1], Suzanne A. Ligthart[4]

1. Department of Primary care and Medical Ethics, Academic Medical Center Amsterdam, Meibergdreef 9, 1100 DD, Amsterdam, the Netherlands
2. Department of Neurology, Academic Medical Center Amsterdam, Meibergdreef 9, 1100 DD, Amsterdam, the Netherlands
3. Department of Neurology, Donders Centre for Brain, Behaviour and Cognition, Radboud University Medical Center, Geert Grooteplein 10, 6525 GA, Nijmegen, the Netherlands
4. Department of Primary and Community Care, Radboud Institute for Health Sciences, Radboud University
Medical Center, Geert Grooteplein 21, 6525 EZ Nijmegen, the Netherlands

Correspondence to: E.P. Moll Van Charante; Academic Medical Center Amsterdam, Meibergdreef 9, 1100 DD, Amsterdam, the Netherlands; Phone, +31-20-5667462; E-mail, e.p.mollvancharante@amc.uva.nl

For peer review only

Abstract

Objectives: Sixty-five percent of older people have hypertension, but little is known about their preferences and concerns regarding hypertension management. Guidelines on hypertension lack consensus on how to treat older people without previous cardiovascular disease. This asks for explicit consideration of patient preferences in decision making. Therefore, the aim of this study was to explore older peoples' experiences, preferences, concerns and perceived involvement regarding hypertension management.

Design: Qualitative interview study.

Setting: Participants were selected from 11 general practitioner (GP) practices in the Netherlands, and purposively sampled until data saturation was achieved. Semi-structured interviews were conducted, audio recorded and analyzed by two researchers using thematic analysis.

Participants: Fifteen community dwelling older people aged 74-93 years with hypertension and without previous cardiovascular disease participated.

Results: Interviewees rarely started the conversation about hypertension management with their GP, although they did have concerns. Reasons for not discussing the subject included low priority of hypertension concerns, reliance on GPs or trust in GPs to make the right decision on their behalf. Also, interviewees anticipated regret of reducing medication, fearing vascular incidents. Interviewees would like to discuss tailoring treatment to their needs, deprescription of medication and ways to reduce side effects. They expected GPs to be more transparent on treatment effects.

Conclusion: Older people describe having little involvement in hypertension management, although they have several concerns. Since GPs are also known to be hesitant to bring up this subject, we signal a conspiracy of silence about antihypertensive medication. Through breaking this silence, GPs can facilitate shared decision-making on hypertension management and better tailored care.

Keywords: hypertension management, older people, primary care, shared decision-making conspiracy of silence

For peer review only

Strengths and limitations of this study:

- Interviews were performed at the participants' homes, enabling them to speak freely about their experiences, wishes and concerns.
- A diverse population of older persons was interviewed.
- Few participants experienced side effects of their medication.
- Information on decision making at the very start of hypertension treatment was limited, due to recall bias.

Funding

This research was supported by the Netherlands Organisation for Health Research and Development (grant number 839110003). The funders had no role in study design, methods, subject recruitment, data collection and analysis, interpretation of data, preparation of the manuscript or the decision to publish.

Competing interests

The Authors declare that there are no conflicts of interest.

For peer review only

Introduction

General practitioners consider hypertension management for older people challenging.[1] For people aged 70 years and over, primary prevention guidelines on cardiovascular disease lack consensus on (de)prescribing antihypertensive medication (AHM). Treatment decisions are further complicated by multi-morbidity, shorter life-expectancy, functional decline, and attenuating benefit-to-harm ratios.[2-5] These uncertainties underline the need for patient involvement in decision making, to tailor care.[5] General practitioners (GPs) wish to take into account patients' preferences and circumstances. However, in everyday practice these are not always optimally addressed and are generally not discussed.[1, 6, 7]

Studies on lay perspectives on blood pressure have shown a gap between patients' and doctors' understanding of hypertension and the need for antihypertensive medication, calling for acknowledgement of these differences and engagement of patients.[8] At present, little is known about older patients' involvement in and preferences on hypertension management in primary care. This is surprising since 65% of older people have hypertension and 82% of them are using antihypertensive medication.[9] Previous studies showed that older people wish to be involved in decision-making about their medical conditions, and expect their GP to consider their personal preferences, situation and concerns in decision-making.[10, 11] However, GPs less often appear to apply shared decision-making in consultations with older patients.[12]

Insight in older patients' experiences regarding hypertension management can support GPs in involving patients in decision making and thus in tailoring hypertension management. With this study we aimed to explore older peoples' experiences with AHM, how they perceive their involvement in hypertension management, and what their preferences and concerns regarding hypertension management are.

Methods

Participants

For this qualitative interview study, community dwelling hypertensive persons aged 70 years or older were recruited from GP practices. We included participants without a history of cardiovascular disease (CVD) because people who experienced CVD may have different motivations for preventing future CVD and taking preventative medication, compared to people without CVD. To ensure diversity, GPs were asked to purposively select older persons based on age, gender, educational level, geographical region, urbanization, functional abilities and experiences with adverse effects of antihypertensive medication. GPs recruited a maximum of two patients per practice. During the iterative process of interviewing and analysing, new GPs were asked to recruit patients with specific characteristics that were, so far, underrepresented in our sample. All participants who agreed to have their contact details passed on to the researchers signed written informed consent and participated. A gift voucher (€10) was offered to participants as compensation for their time.

Data collection

For peer review only

Semi-structured interviews with use of a topic list were held at the participants' homes by LR, EB or both between January and March 2018. Interviews were conducted in Dutch language. In 2018, LR and EB were both female GP trainees who had worked a year in a GP practice and with experience in qualitative and quantitative research. To avoid participants from feeling hesitant to report criticism about GPs, the interviewers introduced themselves as researchers and emphasized that results were confidential and would not be disclosed to the participants' GP. The interview started after introductions, small talk and reiterating the research topic and goal. All participants were interviewed on their views on hypertension, reasons to (not) treat it, the experienced and desired role of the GP, and their own role in treatment decision making. The topic list was modified during the study period, as new themes arose during the first interviews. The interview topic list is presented in Table 1. Once data saturation was achieved (after 15 interviews) no new participants were recruited. Field notes were made after each interview. The duration of the interviews ranged from 20 to 90 minutes.

Analysis

All interviews were audio recorded and transcribed verbatim. Qualitative analysis of the data was performed following the six phases of thematic analysis according to Braun and Clarke[13]:

1) First, two authors (LR and EB) familiarized themselves with the collected data by transcribing the interviews verbatim and repeatedly listening to and reading the data.
2) LR and EB coded the first six interviews independently by systematically going through the data, creating initial codes. After comparing three independently coded interviews, codes were merged. The next three interviews were coded independently, and compared, resulting in a new set of codes. The other interviews were coded by one author using these codes and checked by the other. The coding was compared and discussed until agreement was found.
3) Identified codes were sorted into potential themes which were discussed by the authors (LR, EB, JP, EMC, SL) until consensus about potential themes was reached.
4) Potential themes were reviewed, and thematic maps were made. The collected data was re-read to make sure the thematic map was representing the data set. Whether data saturation was reached was discussed.
5) The resulting themes were refined and a narrative of the found data was considered and discussed by LR, EB, JP, EMC, SL.
6) Final analysis was performed, and illustrative examples were selected and translated into English, in order to answer the research question and compare our analysis to existing literature.

Analysts were GP (trainee) (LR, EB, EMC, SL), neurologist (ER), and/or anthropologist (LR, JP). For coding and analysis MAXQDA Plus 12 (version 12.2.1) was used. Reporting of our study is in accordance with the consolidated criteria for reporting qualitative research (COREQ).[14]

Ethical considerations

Official approval of this study was waived by the Medical Ethics Review Committee of the Academic Medical Centre in Amsterdam (reference W18_004#18.015), the Netherlands.

For peer review only - http://bmjopen.bmj.com/site/about/guidelines.xhtml

Patient and public involvement

There were no patients involved in the development of the research question, the design recruitment to or conduct of the study. This study was specifically designed to bring together the perceptions of hypertensive older patients, to inform hypertension management in general practice.

Results

In total fifteen individuals aged 74-93 years (mean 81) from eleven different GP practices were interviewed. Two lived in senior apartments, all interviewees were living independently. Three received help with groceries and used walking aids. Participants had 3 to 8 (median 4) prescriptions, with 1 to 3 (median 2) types of antihypertensive medication, and level of education ranged from primary school to higher education.(Table S1) Partners of three interviewees were present in the same room during the interview, and occasionally confirmed, contradicted or supplemented the interviewee. Two interviewees had experienced side effects from antihypertensive medication .

In the conducted interviews participants expressed their concerns and preferences regarding hypertension and hypertension management. The results of the interviews were structured in four themes: 'Older peoples' perspectives on hypertension management are not discussed, 'reasons for not discussing needs and preferences regarding hypertension management', 'concerns and preferences regarding hypertension management', and 'uncertainty about implications of potential choices in hypertension management'.

Older peoples' perspectives on hypertension management are not discussed

Interviewees had regular check-ups for their hypertension management, often with a practice nurse. This manuscript focuses on the GP, because interviewees almost exclusively mentioned the GP as the key professional engaged in their hypertension care. The GP takes decisions on antihypertensive medication, and participants consider the role of the practice nurse solely for check-up, follow-up and plain advice. In general, participants did not discuss their concerns regarding hypertension management with their GPs or felt barriers to do so. When directly asked why, interviewees expressed that there was no need to discuss the subject, or that they experienced barriers to discuss issues on hypertension with their GP. During the interviews however, all participants reported concerns which they did not express to their GP. In general, they expected (and waited for) the GP to start the conversation about their preferences and concerns regarding hypertension management. One participant who felt reluctant taking medication reported feeling extremely relieved when her GP suggested to reduce her antihypertensive medication.

'Then she [the GP] said: "why don't we try to reduce the medication?" At that moment I could hug her, I would never dare to ask this myself (…), but if she wants to!' (P6)

There were four key reasons why older persons did not start the conversation about hypertension management with their GP, and four reasons why they desired such a discussion.

For peer review only - http://bmjopen.bmj.com/site/about/guidelines.xhtml

Reasons for not discussing needs and preferences regarding hypertension management

Hypertension is not a priority for older people

When participants experience no symptoms of hypertension or side effects of treatment, they generally did not seem to bother much about hypertension or antihypertensive medication (AHM). Interviewees felt hypertension is part of normal aging, and other diseases or circumstances were more important to them. Taking AHM daily was part of their routine and did not seem to burden these participants.

‘I am not bothered by it [hypertension], so I never think about it. I just never think about it’ (P7)

Relianceon the GP

Fear of jeopardizing the relation with the GP, on whose care they relied for all other (medical) problems, was a barrier to express concerns regarding AHM.

‘You feel dependent on them. You cannot fight with them, because what can you do, it is not easy to change or find another GP (…) so your dependency is bigger than I anticipated.’ (P5)

Also, the perceived authority of the GP formed a barrier to discuss any doubts about AHM. Some interviewees felt that it would be inappropriate to question the GP’s judgement.

‘I don’t want to do that [discuss reducing medication], because then I disregard my GP’s advice, my GP expects me to follow her advice’ (P1)

The GP knows best

Another reason to remain silent was trust in the GP and his or her expertise. Interviewees were convinced that the GP would continuously monitor their medication and trusted him to start the conversation if this was indicated.

‘I just assume that if he thinks I need another pill, that he knows what is best for me’ (P2)

The long-term relation with the GP enforced this trust in the GP’s expertise.

‘I thought, well, the doctor is probably right, who am I, I didn’t study for it, so he probably knows, because he knows me already for a long time’. (P2)

Interviewees found it difficult to truly understand the consequences of risk (reduction) for their personal situation and to interpret that information. This was a barrier to making decisions and discussing doubts with their GP. The uncertainty made interviewees rely on the advice of their GP; the expert.

‘I would like to know [statistics on risk reduction], but I would like to get an answer which says something about my own risk, but there is no such answer, that makes it difficult.’ (P5)

Anticipated regret and avoiding responsibilisation

For peer review only

Interviewees were aware of the threats of hypertension. They feared CVD, particularly cerebrovascular accidents, since it often comes with disability, loss of independence and loss of social contacts. One of the strongest motivators not to express concerns about drug treatment was anticipated regret of developing CVD, when reducing medication.

‘I don’t want to have a stroke (…). I have worked as an activity coordinator in nursing homes. I have seen the results of strokes, I don’t want to experience that.’ (P10)

In addition, actively initiating change in hypertension management felt like taking responsibility for the outcome. Following doctor’s advice, and ignoring concerns about hypertension management was a way to avoid active decision making and responsibility for one’s own situation.

‘I am not going to be stubborn, because if something would happen, it would be my own fault.’ (P2)

In the same line, fear of what might happen kept interviewees from wanting to learn about the details and risks of their condition.

‘No, I don’t want that [more explanation of the doctor about AHM], no. I mean, if he explains to me what will happen if I don’t take them [medication] I would feel uneasy, I think.’ (P7)

Concerns and preferences regarding hypertension management

<u>Wish to reduce antihypertensive medication (AHM)</u>

Even if they did not discuss this with their GP, when asked the interviewees showed interest in reducing medication and had questions about their use of medication. First, there was a wish to minimize overall medication use. Second, participants wondered whether (some of) the medication could be deprescribed, after a certain period of adequately controlled blood pressure. They were curious to find out if hypertension would reappear after stopping medication.

‘My blood pressure is always good, my question is, is that because of the medication? Or maybe I do not need the medication?’ (P14)

<u>Wish to consider one’s age and situation</u>

Taking into account one’s age and situation when deciding on continuation of AHM was considered important. For instance, a participant expressed the concern that his GP would not (sufficiently) adjust his blood pressure levels to his older age:

‘Sometimes I feel that they think you are still a young guy, because you have to reach certain [blood pressure] levels, which might not be relevant anymore for older people.’ (P4

Some participants thought that relatively high levels of stress in the past might have contributed to increased blood pressure levels at the start of their treatment and wondered whether improvements in their wellbeing could have had beneficial effects, allowing for deprescription of AHM.

For peer review only

'"Your blood pressure is too high", well that was based on certain levels, it is possible, I don't have knowledge about that (…), but I keep thinking that it was a particular moment'. (P14

Side effects

Fatigue, fogginess, dizziness, staggering gait, polyuria, nausea, burning sensation in the stomach and reduced exercise tolerance were side effects of AHM that participants reported. These side effects reduced quality of life and resulted in the wish to re-evaluate the benefits and harms of continuing or stopping AHM. For some, this was a reason to discuss hypertension treatment with their GP.

'If it stays like it is at the moment, I think I will accept it. But that burn in your stomach and the feeling that you have to throw up, I don't like that, that was not acceptable for me. It needs to be in my opinion within reason, these side effects. If it becomes too much, I won't accept it. I would then say: "I don't want that drug anymore".' (P15)

Lack of transparency in GPs' considerations

The interviewees who experienced side effects of AHM learned about the uncertainty of their GP regarding hypertension management, during their struggle to find a balance between prevention of CVD and side effects. Despite the knowledge that GPs do not know the optimal blood pressure targets in older age, and thus the right treatment, one of them felt reluctant to discuss his own doubts with the GP. Still, he would have appreciated it if his GP had shown and shared more of his uncertainty, to make room for alternative outcomes (instead of blood pressure target as the only goal).

'They really don't know. They have statistics, but whether you meet those or not, they don't know, they just don't know. I think they should be more transparent about that.' (P5

Uncertainty about implications of potential choices

Overarching the aforementioned themes is the uncertainty of implications of choices regarding hypertension management. Interviewees were ambivalent about deciding which of their concerns and preferences were most important and should be acted upon.

'It [taking antihypertensive medication] is a compromise. (…) Of course, if it would be better [to take more medication] and if it lowers my risk I would accept it. I wouldn't be happy about it. I would be reluctant [to take medication] but it would be necessary, it would be better because you would lower your risks.' (P3)

The final thematic map is shown in Figure 1.

Discussion

For peer review only

Older people do not feel sufficiently involved in decision making about hypertension management in general practice, while they do have many unexpressed concerns. Reasons not to raise these concerns are the low priority compared to other complaints or diseases, being reliant on their GP, trust in their GP to make the right decision on their behalf, and anticipated regret of incident cardiovascular disease (CVD) in absence of treatment. Participants could benefit from overcoming these barriers but wait for their GP to bring up the subject. They often have the wish to reduce medication, to tailor management to their individual situation, to avoid side effects and to receive transparent information on expected treatment effects. Lastly, participants experienced ambivalence regarding their concerns and preferences in hypertension management, hampering their perceived capacity for decision making on their own situation.

Strengths of this study include that interviews were performed at the participants' homes, by researchers who were independent of their GPs. This way we enhanced trust and enabled a diverse population of older persons to speak freely about their experiences, wishes and concerns. Because of the background of the authors (GPs and anthropologists), the analyses had a pragmatic but open character, looking for themes that may inform and improve primary care. The population was divers in terms of age, sex, comorbidities, educational level and geographical region. In terms of dependency and medication use, the population was less divers, with all participants being independent to some extent and taking multiple medications. With only two participants (recently) experiencing side effects of antihypertensive medication (AHM), this groups representation was small in our study, which may have limited our findings. It was difficult to include persons with side effects from AHM, since most older persons were using AHM for a long time without any problems, with difficulty to recall past episodes of problems or reluctance to discuss this with their GP. It is reported that in adults up to 70 years of age, 85% of those starting AHM experienced side effects,[15] while of long-term AHM users (up to 84 years), 20% reported side effects.[16]
Although we aimed to include people without a history of CVD, two participants probably had had CVD (P9 and P15) judging from the interviews and their medication list (acenocoumarol, acetylsalicylic acid). When selecting participants, we relied on the GP to have assessed the inclusion criteria. However, in our study opinions did not differ from participants with definite absence of CVD in their medical history, particularly not on motivation for preventing future CVD or willingness to use preventative medication. Decision making at the very start of hypertension treatment was hard to recall, limiting the information on treatment initiation. Yet, a large proportion of Dutch older adults have long term hypertension and our results apply to this group.[9]
Our results provide insight into experiences and views of older persons with hypertension, that may apply to other developed countries. Although the next generation of older people may have more difficulty with accepting authority in GPs and less problems with speaking up about their concerns, the complexity of decisions in hypertension management is not likely to change.

<u>A conspiracy of silence</u>
There appears to be a conspiracy of silence, in which both the older patient and the GP do not express their concerns regarding antihypertensive treatment. From previous studies it is known that GPs feel that they hardly involve older persons in decisions on hypertension management.[1, 6, 7] Reasons are time constraints, automated prescriptions, negative emotional impact on patients and

For peer review only

anticipated regret in case of cardiovascular events.[6] This study showed that older people too, have the impression of no or minimal involvement in their hypertension management in primary care. From prior studies little is known about (the perceived) involvement of older people in their hypertension management.[17] This contrasts the wish for greater patient involvement in hypertension management among both GPs and older patients.[1, 6, 7]

Reasons to remain silent

There is uncertainty and ambivalence in prioritizing preferences and concerns regarding antihypertensive medication use among older people in our study. Ambivalence has previously been shown in qualitative studies among older people. Antihypertensive medication can provide a feeling of security regarding prevention of CVD, but older patients also worry about side effects and long-term adverse effects of taking chronic medication,[18-20] are cautious to take preventative medication and prefer minimizing medication-use.[19, 21] Concerns are generally outweighed by the perceived necessity of treatment and their GP's recommendation.[21, 22] In our study side effects were the only reason to discuss alternatives with the GP.

Relianceon the GP was a major barrier that is also recognized in shared decision-making (SDM) in other fields. Patients are in a vulnerable position, and can feel too dependent to bring up concerns in interaction with the doctor.[23] Other barriers to active involvement in decisions were reluctance to bear responsibility for cardiovascular outcomes and anticipated regret, also known from SDM in other fields.[23] It was previously shown that anticipated regret has stronger associations with health behavior than other anticipated negative emotions and risk appraisals.[24]

In our study hypertension and hypertension management were generally given low priority. When taking AHM, participants felt safe in terms of preventing CVD morbidity and mortality. However, older people both overestimate the risks of hypertension and the benefits of medication. E.g. hypertensive people with a mean age of 73 year estimated their stroke risk to be 40% within 5 years, and expected over 50% stroke risk reduction from AHM, while in fact 5 year risk of cardiovascular events for this group is 5-20% and stroke risk reduction from AHM is 24-42%.[25-27] After being shown the actual risks and benefits, a quarter of 75 older patients with hypertension participating in a qualitative study became uncertain about antihypertensive medication use, or would decline to take it.[25] Trust in GPs and AHM seems high, but also unrealistic so participants' choices are often based on false, or at least incomplete information. Concerns about taking medication, side effects and tailoring to the individual situation, could receive different prioritization if patients would be better informed and if GPs would be more transparent on their uncertainty.

Breaking the silence

It is important to break the silence since older patients generally wish to be involved in decision-making about their medical issues, despite the abovementioned reasons to remain silent.[10] GPs also value patient involvement in decision making, especially since guidelines leave room for discussion.[6, 28] By breaking the silence, GPs could guide older patients in their ambivalent feelings about AHM, and improve personalized care. For example, once started, lifelong use of AHM is the current standard of care. However, a systematic review showed that after 2 years of withdrawal of AHM, hypertension did not return in 26% of patients (mean age 41 to 76 years). This justifies an AHM withdrawal attempt in well controlled patients, if they wish to do so.[29] In addition, more

For peer review only - http://bmjopen.bmj.com/site/about/guidelines.xhtml

transparency could reduce misinterpretations and lower the threshold to start discussing factors important to patients in hypertension management.

Heterogeneity exists in how older people want to be involved in decision-making. Older patients with multi-morbidity and healthcare professionals agreed that SDM in older patients requires a continuous dialogue between professional and patient.[30] Contextual factors for receiving information, such as having enough time and having a good relationship with professionals involved, are considered of great importance.[31] Elements in decision making unique to older people are the opportunity for input from trusted others and discussion of decisions' impacts on patients daily lives.[32]

Older persons generally do not start the dialogue themselves; they wait for their GP to do so. In our study, and also in SDM in other fields of medicine, older patients experience barriers to participate in decision making about their care.[33] Since most barriers are related to the GP, including the authority ascribed to them as well as time constraints, we recommend that GPs take the initiative to break the silence.[22]

Implication for practice

To break the conspiracy of silence, GPs should explicitly and repeatedly discuss hypertension treatment with their patients on chronic antihypertensive medication. This is the only way to come to true shared decision-making about hypertension treatment and may lead to adaptation and potentially cessation of antihypertensive medication in those for whom the benefits not clearly outweigh the burden.

For peer review only

Ethical approval

Official approval of this study was waived by the Medical Ethics Review Committee of the Academic Medical Centre in Amsterdam (reference W18_004#18.015), the Netherlands.

Financial disclosure

No financial disclosures were reported by the authors of this manuscript.

Acknowledgements

We sincerely thank all individuals who participated in this qualitative study, and all general practitioners and their assistants who selflessly recruited participants.

Author contributions

EvB, LR, JP, ER, EMvC and SL contributed to the conception or design of the work. EvB and LR performed the interviews. EvB, LR, JP, ER, EMvC and SL contributed to the acquisition, analysis, or interpretation of data for the work. EvB drafted the manuscript. EvB, LR, JP, ER, EMvC and SL critically revised the manuscript and gave final approval and agree to be accountable for all aspects of work ensuring integrity and accuracy.

Data sharing statement

Data underlying our findings cannot be made publicly available for ethical reasons; public availability would compromise our participants' privacy. Data requests may be sent to the corresponding author at e.p.mollvancharante@amc.uva.nl

Licence statement

I, the Submitting Author has the right to grant and does grant on behalf of all authors of the Work (as defined in the below author licence), an exclusive licence and/or a non-exclusive licence for contributions from authors who are: i) UK Crown employees; ii) where BMJ has agreed a CC-BY licence shall apply, and/or iii) in accordance with the terms applicable for US Federal Government officers or employees acting as part of their official duties; on a worldwide, perpetual, irrevocable, royalty-free basis to BMJ Publishing Group Ltd (“BMJ”) its licensees and where the relevant Journal is co-owned by BMJ to the co-owners of the Journal, to publish the Work in BMJ Open and any other BMJ products and to exploit all rights, as set out in our licence.

The Submitting Author accepts and understands that any supply made under these terms is made by BMJ to the Submitting Author unless you are acting as an employee on behalf of your employer or a postgraduate student of an affiliated institution which is paying any applicable article publishing charge (“APC”) for Open Access articles. Where the Submitting Author wishes to make the Work available on an Open Access basis (and intends to pay the relevant APC), the terms of reuse of such

For peer review only

Open Access shall be governed by a Creative Commons licence – details of these licences and which Creative Commons licence will apply to this Work are set out in our licence referred to above.

Figure caption: Figure 1. Final thematic map
Figure legend: Graphical display of the four themes of this qualitative interview study, their subthemes and mutual relations.
HT, hypertension; GP, general practitioner; AHM, antihypertensive medication

For peer review only

References

1. Jansen J, McKinn S, Bonner C, et al. General Practitioners' Decision Making about Primary Prevention of Cardiovascular Disease in Older Adults: A Qualitative Study. PLoS One. 2017;12(1):e0170228.
2. Perk J, De Backer G, Gohlke H, et al. European guidelines on cardiovascular disease prevention in clinical practice (version 2012) : the fifth joint task force of the European society of cardiology and other societies on cardiovascular disease prevention in clinical practice (constituted by representatives of nine societies and by invited experts). Int J Behav Med. 2012;19(4):403-88.
3. Streit S, Verschoor M, Rodondi N, et al. Variation in GP decisions on antihypertensive treatment in oldest-old and frail individuals across 29 countries. BMC Geriatr. 2017;17(1):93.
4. Weiss J, Freeman M, Low A, et al. Benefits and Harms of Intensive Blood Pressure Treatment in Adults Aged 60 Years or Older: A Systematic Review and Meta-analysis. Ann Intern Med. 2017;166(6):419-29.
5. Jansen J, McKinn S, Bonner C, et al. Systematic review of clinical practice guidelines recommendations about primary cardiovascular disease prevention for older adults. BMC Fam Pract. 2015;16:104.
6. van Middelaar T, Ivens SD, van Peet PG, et al. Prescribing and deprescribing antihypertensive medication in older people by Dutch general practitioners: a qualitative study. BMJ Open. 2018;8(4):e020871.
7. Clinch M, Benson J. Making information 'relevant': general practitioner judgments and the production of patient involvement. Soc Sci Med. 2013;96:104-11.
8. Marshall IJ, Wolfe CD, McKevitt C. Lay perspectives on hypertension and drug adherence: systematic review of qualitative research. BMJ. 2012;345:e3953.
9. Nwankwo T, Yoon SS, Burt V, Gu Q. Hypertension among adults in the United States: National Health and Nutrition Examination Survey, 2011-2012. NCHS Data Brief. 2013(133):1-8.
10. Bastiaens H, Van Royen P, Pavlic DR, et al. Older people's preferences for involvement in their own care: a qualitative study in primary health care in 11 European countries. Patient Educ Couns. 2007;68(1):33-42.
11. Rotar-Pavlic D, Svab I, Wetzels R. How do older patients and their GPs evaluate shared decision-making in healthcare? BMC Geriatr. 2008;8:9.
12. Meijers MC, Noordman J, Spreeuwenberg P, et al. Shared decision-making in general practice: an observational study comparing 2007 with 2015. Fam Pract. 2019;36(3):357-64.
13. Braun VC, V. . Using thematic analysis in psychology. . In: Clarke V, editor. Qualitative Research in Psychology2006. p. 77-101.
14. Tong A, Sainsbury P, Craig J. Consolidated criteria for reporting qualitative research (COREQ): a 32-item checklist for interviews and focus groups. Int J Qual Health Care. 2007;19(6):349-57.
15. Tedla YG, Bautista LE. Drug Side Effect Symptoms and Adherence to Antihypertensive Medication. Am J Hypertens. 2016;29(6):772-9.
16. Bardage C, Isacson DG. Self-reported side-effects of antihypertensive drugs: an epidemiological study on prevalence and impact on health-state utility. Blood Press. 2000;9(6):328-34.
17. Butterworth JE, Campbell JL. Older patients and their GPs: shared decision making in enhancing trust. Br J Gen Pract. 2014;64(628):e709-18.
18. Moen J, Bohm A, Tillenius T, et al. "I don't know how many of these [medicines] are necessary.." - a focus group study among elderly users of multiple medicines. Patient Educ Couns. 2009;74(2):135-41.
19. Benson J, Britten N. Patients' decisions about whether or not to take antihypertensive drugs: qualitative study. BMJ. 2002;325(7369):873.
20. Clyne B, Cooper JA, Boland F, et al. Beliefs about prescribed medication among older patients with polypharmacy: a mixed methods study in primary care. Br J Gen Pract. 2017;67(660):e507-e18.

For peer review only

21. Gale NK, Greenfield S, Gill P, et al. Patient and general practitioner attitudes to taking medication to prevent cardiovascular disease after receiving detailed information on risks and benefits of treatment: a qualitative study. BMC Fam Pract. 2011;12:59.
22. Modig S, Kristensson J, Troein M, et al. Frail elderly patients' experiences of information on medication. A qualitative study. BMC Geriatr. 2012;12:46.
23. van Laarhoven HW, Henselmans I, de Haes JH. To treat or not to treat: who should decide? Oncologist. 2014;19(4):433-6.
24. Brewer NT, DeFrank JT, Gilkey MB. Anticipated regret and health behavior: A meta-analysis. Health Psychol. 2016;35(11):1264-75.
25. Cranney M, Warren E, Walley T. Hypertension in the elderly: attitudes of British patients and general practitioners. J Hum Hypertens. 1998;12(8):539-45.
26. Ligthart SA, Moll van Charante EP, Van Gool WA, Richard E. Treatment of cardiovascular risk factors to prevent cognitive decline and dementia: a systematic review. Vasc Health Risk Manag. 2010;6:775-85.
27. Beer C, Alfonso H, Flicker L, et al. Traditional risk factors for incident cardiovascular events have limited importance in later life compared with the health in men study cardiovascular risk score. Stroke. 2011;42(4):952-9.
28. Wetzels R, Geest TA, Wensing M, et al. GPs' views on involvement of older patients: an European qualitative study. Patient Educ Couns. 2004;53(2):183-8.
29. van der Wardt V, Harrison JK, Welsh T, et al. Withdrawal of antihypertensive medication: a systematic review. J Hypertens. 2017;35(9):1742-9.
30. van de Pol MH, Fluit CR, Lagro J, et al. Expert and patient consensus on a dynamic model for shared decision-making in frail older patients. Patient Educ Couns. 2016;99(6):1069-77.
31. Robben S, van Kempen J, Heinen M, et al. Preferences for receiving information among frail older adults and their informal caregivers: a qualitative study. Fam Pract. 2012;29(6):742-7.
32. Price EL, Bereknyei S, Kuby A, et al. New elements for informed decision making: a qualitative study of older adults' views. Patient Educ Couns. 2012;86(3):335-41.
33. Bane C, Hughes CM, Cupples ME, McElnay JC. The journey to concordance for patients with hypertension: a qualitative study in primary care. Pharm World Sci. 2007;29(5):534-40.

For peer review only

Table 1. Interview topic list

**Topic**	Subtopic
**Participant characteristics**	Demographics, including medical history and medication use
**Hypertension and antihypertensive medication**	Frequency of hypertension visits at the GP practice
	Topics discussed during hypertension visits at the GP practice: medication use, side effects, lifestyle.
	Experience with hypertension, antihypertensive medication (use), and lifestyle (changes) since hypertension was first identified.
	Attitudes towards hypertension, antihypertensive medication (use), lifestyle, and necessity of treatment
	Experiences with and attitudes towards deprescribing antihypertensive medication
**Shared decision-making**	Received information on hypertension, risk and risk reduction from antihypertensive medication and lifestyle during (hypertension) visits at the GP practice
	Experience of being involved in decision making and receiving tailored information and care.
	Views on involvement and tailoring information and care by GP
	Experience of trust in the GP and in hypertension management in primary care
	Experiences of GPs' uncertainties in hypertension management in older persons
	Experience of and views on necessity, barriers and facilitators to discuss hypertension management with the GP
**Personal situation and preferences**	Experience of and views on consideration of personal circumstances and age in decision making in hypertension management
	Experienced effects of one's personal situation on views on hypertension and its management
	Experienced prioritization of facets of hypertension and its management
	Experienced effect of chronic hypertension and long-term medication use on views on hypertension and its management
**Future perspectives**	Wish to change things regarding hypertension management: lifestyle, antihypertensive medication use, or involvement in decision making with the GP.

GP, general practitioner

For peer review only

For peer review only

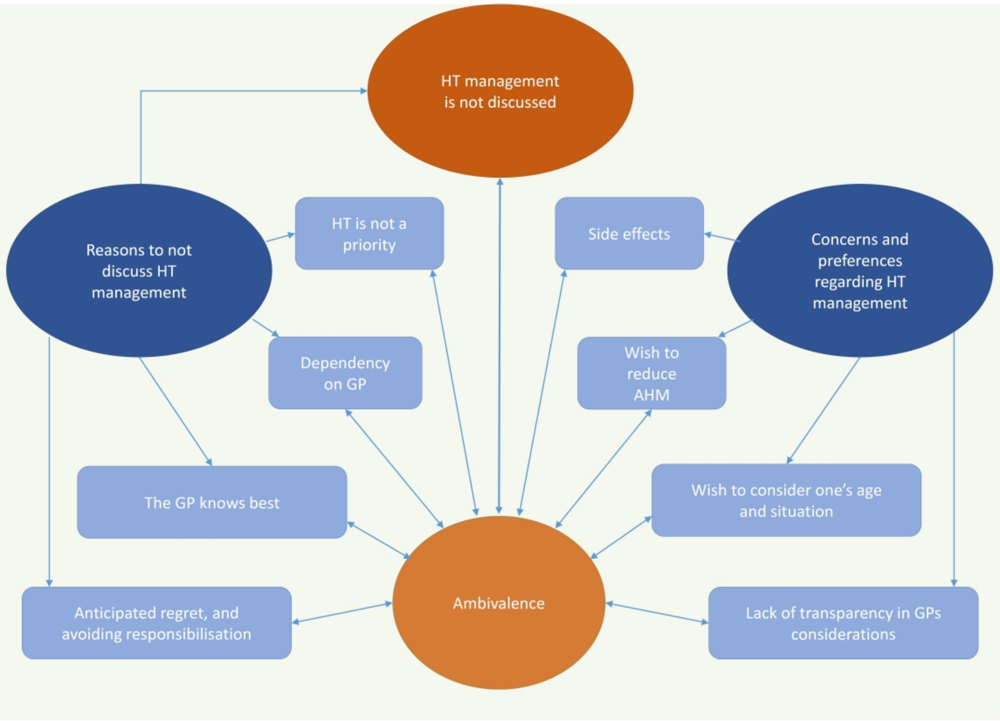

Figure caption: Figure 1. Final thematic map
Figure legend: Graphical display of the four themes of this qualitative interview study, their subthemes and mutual relations.
HT, hypertension; GP, general practitioner; AHM, antihypertensive medication

481x346mm (300 x 300 DPI)

Supplementary file

Hypertension management: experiences, wishes and concerns among older people – a qualitative study

Table S1. Characteristics of participants

Gender	Age (years)	Multimorbidity [a]	Living location [b]	Educational level	Dependency [c]	Number of AHM	Total number of prescriptions [d]
**Female**	74	Yes	Small city	Middle	Independent	3	8*
**Male**	74	No	Village	High	Independent	2	2
**Female**	75	Yes	Small city	Unknown	Independent	1	3*
**Female**	75	No	Village	High	Independent	2	4
**Female**	77	Yes	Small city	Unknown	Independent	3	5
**Female**	79	Yes	Village	High	Independent	2	4
**Female**	80	Yes	Town	High	Independent	2	7*
**Male**	81	No	Small city	High	Independent	1	3
**Male**	82	Yes	City	Primary	Independent	3	7
**Male**	84	No	Village	High	Independent	3	4
**Male**	84	No	Village	Middle	ADL independent	2	6
**Female**	86	Yes	Town	High	Independent	2	4
**Female**	86	Yes	City	Middle	ADL independent	3	7
**Female**	87	No	Village	Middle	Independent	1	2
**Female**	93	No	City	Primary	ADL independent	2	3

AHM, antihypertensive medication; ADL, activities of daily living; T2DM, type 2 diabetes mellitus.
a. Multimorbidity (y/n), defined as having at least two chronic conditions, in addition to hypertension. Medical history based on self-report during interview.
b. City, >100,000 inhabitants; Small city, 10,000-100,000 inhabitants; Town 5,000-10,000 inhabitants; Village <5,000 inhabitants
c. Independent: able to take care of ADL and doing household chores/ run errands. ADL independent: able to take care of ADL, but dependent on doing household chores/ run errands.
d. * indicates the participant used a medication box, no one used medication rolls.

For peer review only

COREQ (COnsolidated criteria for REporting Qualitative research) Checklist

A checklist of items that should be included in reports of qualitative research. You must report the page number in your manuscript where you consider each of the items listed in this checklist. If you have not included this information, either revise your manuscript accordingly before submitting or note N/A.

Topic	Item No.	Guide Questions/Description	Reported on Page No.
**Domain 1: Research team and reflexivity**			
Personal characteristics			
Interviewer/facilitator	1	Which author/s conducted the interview or focus group?	4
Credentials	2	What were the researcher's credentials? E.g. PhD, MD	4,5
Occupation	3	What was their occupation at the time of the study?	4,5
Gender	4	Was the researcher male or female?	4,5
Experience and training	5	What experience or training did the researcher have?	4,5
Relationship with participants			
Relationship established	6	Was a relationship established prior to study commencement?	4,5
Participant knowledge of the interviewer	7	What did the participants know about the researcher? e.g. personal goals, reasons for doing the research	NA
Interviewer characteristics	8	What characteristics were reported about the inter viewer/facilitator? e.g. Bias, assumptions, reasons and interests in the research topic	NA
**Domain 2: Study design**			
Theoretical framework			
Methodological orientation and Theory	9	What methodological orientation was stated to underpin the study? e.g. grounded theory, discourse analysis, ethnography, phenomenology, content analysis	5
Participant selection			
Sampling	10	How were participants selected? e.g. purposive, convenience, consecutive, snowball	4
Method of approach	11	How were participants approached? e.g. face-to-face, telephone, mail, email	4
Sample size	12	How many participants were in the study?	6
Non-participation	13	How many people refused to participate or dropped out? Reasons?	4
Setting			
Setting of data collection	14	Where was the data collected? e.g. home, clinic, workplace	4
Presence of non-participants	15	Was anyone else present besides the participants and researchers?	4,5
Description of sample	16	What are the important characteristics of the sample? e.g. demographic data, date	6
Data collection			
Interview guide	17	Were questions, prompts, guides provided by the authors? Was it pilot tested?	4,5
Repeat interviews	18	Were repeat inter views carried out? If yes, how many?	na
Audio/visual recording	19	Did the research use audio or visual recording to collect the data?	4,5
Field notes	20	Were field notes made during and/or after the inter view or focus group?	4,5
Duration	21	What was the duration of the inter views or focus group?	4,5
Data saturation	22	Was data saturation discussed?	5
Transcripts returned	23	Were transcripts returned to participants for comment and/or	na

For peer review only

Topic	Item No.	Guide Questions/Description	Reported on Page No.
		correction?	
**Domain 3: analysis and findings**			
Data analysis			
Number of data coders	24	How many data coders coded the data?	5
Description of the coding tree	25	Did authors provide a description of the coding tree?	na
Derivation of themes	26	Were themes identified in advance or derived from the data?	5
Software	27	What software, if applicable, was used to manage the data?	5
Participant checking	28	Did participants provide feedback on the findings?	5
Reporting			
Quotations presented	29	Were participant quotations presented to illustrate the themes/findings? Was each quotation identified? e.g. participant number	6 - 9
Data and findings consistent	30	Was there consistency between the data presented and the findings?	6 - 9
Clarity of major themes	31	Were major themes clearly presented in the findings?	6 - 9
Clarity of minor themes	32	Is there a description of diverse cases or discussion of minor themes?	6-9

Developed from: Tong A, Sainsbury P, Craig J. Consolidated criteria for reporting qualitative research (COREQ): a 32-item checklist for interviews and focus groups. *International Journal for Quality in Health Care*. 2007. Volume 19, Number 6: pp. 349 – 357

Once you have completed this checklist, please save a copy and upload it as part of your submission. DO NOT include this checklist as part of the main manuscript document. It must be uploaded as a separate file.

For peer review only
